# The Discomfort of Riding Shotgun – Why Many People Don’t Like to Be Co-driver

**DOI:** 10.3389/fpsyg.2020.584309

**Published:** 2020-11-16

**Authors:** Sandra Ittner, Dominik Mühlbacher, Thomas H. Weisswange

**Affiliations:** ^1^Wuerzburg Institute for Traffic Sciences GmbH, Veitshöchheim, Germany; ^2^Honda Research Institute Europe GmbH, Offenbach, Germany

**Keywords:** information processing, cognition, passenger, comfort, feedback-loop model, situation awareness, risk assessment, autonomous driving

## Abstract

This work investigates which conditions lead to co-driver discomfort aside from classical motion sickness, what characterizes uncomfortable situations, and why these conditions have a negative effect. The automobile is called a “passenger vehicle” as its main purpose is the transportation of people. However, passengers in the car are rarely considered in research concerning driving discomfort. The few studies in this area focus on driver discomfort, automated vehicles, or driver assistant systems. An earlier public survey indicated that discomfort is also a relevant problem for co-drivers. In this paper, these results are confirmed and extended through an online questionnaire with *N* = 119 participants and a detailed follow-up interview study with *N* = 24 participants was conducted. The results of the online questionnaire show that co-driver discomfort is a widespread problem (88%). The interviews indicate that the driving style is one factor contributing to co-driver discomfort, in particular close following or fast driving. In those situations, participants experienced a feeling of being exposed, which additionally contributed to their discomfort. Uncomfortable situations were also perceived as safety critical. A model for possible cognitive origins of discomfort in co-drivers, extending theories from the areas of stress and self-regulation, is developed based on the results. Co-driver discomfort is a common problem, highlighting the relevance of further research on supporting co-drivers. The reported correlations and the proposed model can help to explain the origin of this discomfort. The results provide a foundation for the future design of interventions like human machine interfaces aiming at reducing co-driver discomfort.

## Introduction

The search for literature about discomfort in road vehicles shows a focus on drivers, even though vehicles are intended to transport multiple passengers. Future developments will most likely lead to even higher amounts of passengers in vehicles. Calls for reducing CO_2_ emissions might increase ride sharing solutions for the general population. Such a solution then consequently leads to higher amounts of passengers in cars. Additionally, with higher automation levels of vehicles, even the driver will become a passenger in an automated vehicle. Research about the passengers is very rare, and studies on passenger discomfort are even fewer. However, a survey conducted by an opinion research institute (Innofact Ag for AutoScout24, 2013) showed that 77% of the participants already experienced situations in which they felt uncomfortable as a front-seat passenger. Ellinghaus and Schlag (2001) conducted a survey, which showed that 19% of the participants partially or completely agreed that they are frequently afraid of accidents as a passenger. This indicates that it should be investigated in more detail which factors influence the discomfort of front-seat passengers to derive ideas for interventions, like a human machine interface (HMI), to make their rides more comfortable.

### Definition of the Passenger’s Situation and Role in a Vehicle

In order to identify factors influencing the comfort/discomfort of passengers in a vehicle, it is important to specify what characterizes the role of a passenger. There exist two common definitions. A more general definition of a “passenger” by the Oxford English Dictionary (Stevenson and Lindberg, 2017) is: “a traveler on a public or private conveyance other than the driver, pilot or crew.” In this definition, everyone in a vehicle, who is not concerned with the regular operation, is a passenger. Additionally, it reflects the mostly passive role of passengers in a vehicle. There is also another definition that focuses on the front-seat passenger, who is sometimes called the co-driver. In the Oxford English Dictionary (Stevenson and Lindberg, 2017) the “co-driver” is described as: “a person who shares the driving of a vehicle with another” or (in rally driving) “a person who navigates from the front passenger seat.” In contrast to the first definition, this one specifies the position of the passenger in the car and describes a more active role of the front-seat passenger as sharing the driving task with the driver or supporting the driver during navigation.

Besides the above definitions, the front seat is also characterized by the possibility to monitor the traffic situation without visual obstructions caused by the front seats, or a larger distance to the windscreen. However, the perspective is slightly different from the driver’s visual angle, which might result in different percepts (e.g., distance estimations). It is also possible for the front seat passenger to use the center console and to derive information, for example for navigation.

The dashboard provides additional driving relevant information like velocity or fuel level, but these are usually not easily accessible to the front seat passenger. These devices are mainly designed to show information to the driver. This means that even though the front seat passenger is part of the ride, he/she has only limited control and information about it. Furthermore, the front seat passenger only indirectly receives visual or haptic feedback about the state of the driving task, such as the position of the brake or gas pedal, and therefore has to deal with delayed or biased information. In the following sections, we focus on the front seat passenger because of their greater involvement in the driving task caused by their position in the vehicle. Due to this greater involvement in the driving task, we refer to the front seat passenger as co-driver in the remainder of this work.

### Definition of Discomfort

Similar to emotions, feelings like discomfort are a result of a complex evaluation of stimuli. They signal the motivational significance of internal and external stimuli with respect to current goals and needs (Bower and Cohen, 1982; Lazarus, 1982; Leventhal and Scherer, 1987). For the co-driver, this means that the perceived discomfort signals that something influences their goal of a safe and relaxed ride. Emotions contain reactions on three levels: motoric (muscles, motions), physiological-humoral (CNS, ANS), and subjective-psychological (feelings). An uncomfortable situation can for example lead to sweating or muscle tension. The Oxford English Dictionary (Stevenson and Lindberg, 2017) defines “discomfort” as a feeling of “slight pain” or “to make (someone) feel uneasy, anxious or embarrassed”. The definition highlights that discomfort can arise on a physiological level as in the first part of the definition or on a psychological level as in the second part. Discomfort can therefore be measured in different ways. The definition by the Oxford English Dictionary (Stevenson and Lindberg, 2017) also shows a link to anxiety. Anxiety is a consequence when a dangerous situation is identified. This leads to the possibility that experienced discomfort can also be a consequence of situations that are estimated as dangerous or safety critical by co-drivers. Emotions like anxiety or discomfort are also subjective which means that different stimuli can affect people differently (Drummond et al., 2003). Cosmides and Tooby (2000) described emotions as motivational programs coordinating different behaviors to solve adaptive problems. The fight or flight system will be activated to cope with situations like the avoidance of enemies or the avoidance dangers. Such a coping process could also be relevant for co-drivers experiencing discomfort.

Discomfort is also a possible symptom of the concept of motion sickness, which is already well investigated. Much research addressed conditions and factors leading to motion sickness (Turner and Griffin, 1999a,b; Turner, 1999) as well as individual characteristics, like differences between driver and co-driver (Rolnick and Lubow, 1991) or sex (Koslucher et al., 2015), influencing the susceptibility to motion sickness. Motion sickness is a complex concept with symptoms from the areas of gastrointestinal, central, peripheral, or sopite-related symptoms for example suggested by Gianaros et al. (2001). Motion sickness is caused by primarily physiological mechanisms as explained in many different theories like the sensory conflict/rearrangement theories (Irwin, 1881; Claremont, 1931; Reason, 1978) or other related theories (Bos and van der Bles, 1998; Bles et al., 1998). However, this paper will focus on psychological discomfort caused by cognitive/psychological mechanisms involving the cognitive assessment of external stimuli. Discomfort caused by an impolite or unsympathetic driver is based on more social mechanisms and is not connected to the driving context. Therefore, it is also not targeted in this work.

## Related Work

This section starts by describing two influential cognitive models that can be related to the feeling of discomfort. These will then be mapped to the driving task and used to highlight the potential differences between the driver and the co-driver perspective. Discomfort in the context of driving is a general concept applicable to all participants of a ride including the driver. For example, if someone is tailgating the vehicle, most drivers will feel discomfort. It is likely that factors leading to driver discomfort can also induce co-driver discomfort or produce even higher co-driver discomfort. Therefore, the second part of the section describes related work on driver discomfort following the structure induced by the models. It concludes with a discussion on the relations and differences between driver and co-driver discomfort and an overview of relevant research on the latter.

### Explaining the Development of Discomfort

**FIGURE 1:**
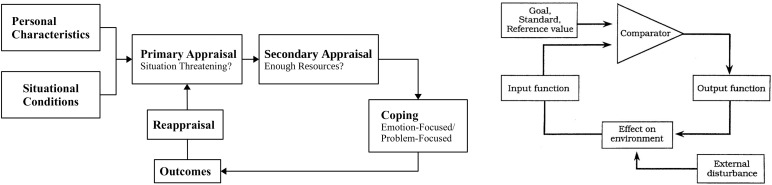
The transactional stress model by Lazarus and Folkman (1984) (left). The feedback-loop model by Carver and Scheier (2002) (right). Copyright 2002 by Sage Pubilcations, Inc.

The ***transactional stress model*** by Lazarus and Folkman (1984) is a cognitive model describing a repeated evaluation of situations regarding their potential threat and the subsequent coping with these situations. The threat of a situation is estimated based on ***situational conditions*** and ***personal characteristics*** (Figure 1 left). Personal characteristics can for example include experiences, personality, or values. Situational conditions are stimuli of the environment with dimensions like intensity, duration, and if they can be controlled or predicted. If a situation is evaluated as threatening or harmful, it will be decided whether there are enough resources to cope with it. The next step would be to decide on a ***coping strategy***. The model states two different ways to do this. One strategy is to actively cope with the situation by addressing the threat (***problem-focused***). The other strategy is more passive and aims at changing the experienced emotions when it is not possible to change or escape the situation (***emotion-focused***). This can be, for example, approached through distraction, avoidance, or denial. Selecting the problem-focused strategy will be more likely when a situation seems controllable, the emotion-focused one will be selected when there is limited influence on a situation. This means that it strongly depends on the situation which coping strategy is chosen (Folkman et al., 1986).

This cognitive model can help to describe the development of driver and co-driver discomfort and why they sometimes evaluate situations differently. In the transactional stress model, the estimation process of a threat is described for general cases and is not directly related to the regulation of a driving task. The ***feedback-loop model*** (Miller et al., 1960; Carver and Scheier, 1998) might be better suited to describe which cognitive processes take place during the regulation of a driving task and where sources of discomfort may lie. The model by Carver and Scheier (1998) (Figure 1 right) generally describes the self-regulation process of human behavior. According to the model, someone has a “reference value,” goal, or a standard on the basis of which incoming perceptions (“input-function”) are compared (“comparator”). If these perceptions differ from the reference value, goal, or standard, humans will show correcting behavior (“output-function”) to adapt their perceptions to the reference value or standard in a looping process. Since humans are bad at estimating absolute values like in the case of distances (Sedgwick, 1986) and velocities (Runeson, 1974), this “reference value” is to be understood more as a subjective judgment. In the next paragraph, this model will be combined with the transactional stress model and parallels between both models will be explained. The main purpose of this combination is to use the more detailed coping process of the transactional model, while the feedback-loop model describes in more detail the regulation of the driving task by the driver. It also shows the relevance of information about the driver’s cognitive state during the co-driver’s regulation process.

If both models are mapped to the driver, he/she will evaluate situations based on ***situational conditions*** and ***personal characteristics.*** In this context, situational conditions can be anything having a negative influence on a relaxed and safe ride, like poor road conditions or a high traffic density. Examples of personal characteristics are driving experience, preferred driving style, or individual personality. The driver influences a situation for example by regulating velocity and distance through iterative adjustments. The driver compares (comparator in feedback-loop, ***primary and secondary appraisal*** in the transactional model) the actual velocity and distance (input-function, ***influencing factors*** with ***situational conditions***) with their preferred velocity and distance (reference value based upon ***personal characteristics***). If there is a discrepancy between actual and preferred velocity and distance, it will be adjusted through a reaction (output function, ***coping strategy***). Since many situations can be controlled by the driver, the main way to cope with them is the active and problem-focused way, for example using the brake or gas pedal to increase the distance to other vehicles or changing the lane by using the steering wheel. The results of these reactions can lead to a change in the environment perceived by the driver. The perception-reaction loop is repeated until the preferred velocity and distance are reached. At each point of the feedback-loop, the driver has information about his/her own cognitive state and direct control of the situation.

In the following two subsections existing research about driver and co-driver discomfort will be presented and discussed following the structure of the processes in the combined transactional stress and feedback-loop model. We will start with ***situational conditions*** and ***personal characteristics*** influencing the estimation of a situation, will then present relevant research about ***coping*** mechanisms and discuss them considering the driving context for driver and co-driver.

### Driver Discomfort

Research on situations in which drivers feel comfortable or uncomfortable often covers ***vehicle*** focused factors of ***situational conditions*** influencing physiological discomfort rather than psychological factors. For example, a study by Qatu (2012) identified noise and vibrations as the main influences on driver comfort, Le et al. (2014) or Hiemstra-van Mastrigt et al. (2017) investigated seating comfort. However, some studies also included psychological discomfort/comfort factors of situations, focusing on the ***environment***, such as road infrastructure (e.g., complex situations like intersections or roundabouts; Cahour, 2008), other road users (e.g., violent driving style; Dorantes et al., 2016) or the weather (e.g., darkness or skidding; Cahour, 2008). One study identified driving tasks like distance keeping during high traffic density as relevant (de Vos et al., 1997). The study by Constantin et al. (2014) investigated various elements in a ***vehicle*** causing discomfort for younger drivers and mapped it to the two dimensions of psychological and physiological discomfort: The seat, the space in the car, and the air condition were mentioned most often for the physical dimension. In the psychological dimension, especially a malfunction of safety relevant elements (e.g., headlights, brakes, or horn) caused discomfort. These situations can be considered as safety critical and uncomfortable since they limit the interaction possibilities with the driving environment.

***Personal Characteristics*** can also influence the conditions under which a situation is considered safety critical by a driver. The relationship between personality traits, driving style, and accident involvement is well investigated (Trimpop and Kirkcaldy, 1997; Sümer, 2003; Oltedal and Rundmo, 2006). A study by Iversen and Rundmo (2001) for example showed that drivers scoring high on sensation seeking, driver anger, and normlessness had a more risky driving style. They were also more frequently involved in near-accidents or crashes (with injuries or vehicle damage). This indicates that drivers scoring high on these personality traits and therefore showing a more risky driving style could have a higher threshold for critical situations and experience discomfort in more critical situations than persons scoring lower on these traits.

The studies named in the last two paragraphs indicate that ***personal characteristics*** and ***situational conditions***, especially of the areas ***vehicle*** and ***environment***, can influence the driver’s estimation of the risk in a situation. Most research is focused on the improvement of driver comfort influenced by technical causes. This could be affected by the circumstance that discomfort caused by environmental factors can be directly controlled and influenced by the driver’s behavior in contrast to most vehicle factors such as the seat or the available space in a vehicle.

### Co-driver Discomfort

Since the co-driver is also part of the ride and interested in a safe and relaxed arrival at a destination, it is likely that the co-driver also evaluates possible threats in a situation. According to the transactional model (Lazarus and Folkman, 1984), he/she would also base these estimations on the same ***situational conditions.*** However, in contrast to the driver, the role of the co-driver or passenger is mostly passive. The co-driver can monitor the traffic situation but has no direct means of intervention. It is also possible for the co-driver to use the center console and to derive information, for example for navigation. The dashboard provides additional driving relevant information like velocity or fuel level, but these are usually not easily accessible to the co-driver. These devices are mainly designed to show information to the driver. The perspective on the environment is slightly different from the driver’s visual angle as well, which might result in different percepts (for example distance estimates). The situational conditions introduced as causing discomfort for the driver could be similar for the co-driver (for example road conditions of the area ***environment*** or a malfunction of headlights of the area ***vehicle***). However, situations that are uncomfortable for the driver could be even more uncomfortable for the co-driver because of his/her special role in the vehicle, caused by differences in available control and information.

According to survey results by the opinion research institute Innofact AG for AutoScout24 GmbH, the ***driver*** can be an additional situational condition. The survey (Innofact Ag for AutoScout24, 2013) focused on co-driver discomfort and asked participants which situations caused it. In this survey, 76% of the participants mentioned that fast driving and close following caused discomfort, followed by false reactions of the driver (60%) and a distracted driver (53%).

There is also relevant work in the area of automated driving, where the “driver” has a passive role. These studies also highlight the influence of the driving style of the automated vehicle on discomfort. In a simulator study by Mühl et al. (2019) it was investigated which driving styles of fully automated vehicles or of human drivers are preferred. The participants showed more positive appraisals (e.g., trust ratings) for a defensive human driving style than for a sporty human driving style or either automated driving styles (defensive or sporty). In another simulator study by Griesche et al. (2016) all participants rejected small safety distances and high accelerations. Similar results were found in the study by de Vos et al. (1997). Participants rated larger time headways as more comfortable during a ride with a fully automated vehicle. A Wizard of Oz study by Yusof et al. (2016) investigated which autonomous driving styles were preferred in a real driving experiment. The results showed that defensive driving styles were generally preferred. Assertive drivers did not prefer their own driving style when experiencing it during a ride with an automated vehicle. These results indicate that it could be uncomfortable for the co-driver when the driver does not follow a defensive driving style, follows with small distances, or accelerates strongly. It is also possible, that the critical values that cause discomfort when being a co-driver might be different from those when being a driver. Unfortunately, most studies about comfort or trust during a fully automated ride are either simulator or Wizard of Oz studies with a human driver simulating the automation. This can reduce the feeling of lacking control or can lead to higher trust in automation compared to realistic conditions. Social factors also do not play a role in research with automated vehicles. The relationship between driver and co-driver could influence whether the co-driver dares to criticize the drivers driving style.

In contrast to the driver, there is another situational condition for the co-driver: The ***cognitive state of the driver***. This cognitive state is also unknown to co-drivers and can subsequently influence the estimation of the situation’s criticality. There can be uncertainty about whether the driver has his/her attention on the critical situation, whether he/she estimates the situation as critical as the co-driver, or how the driver will react to this situation. The role of the cognitive state of the driver is comparable to the system state in automated vehicles. The latter often informs the driver about the current state, detected objects, or planned maneuvers through HMIs creating situation awareness in the driver. This can make it easier for the driver to decide when to take back control of the vehicle (e.g., Naujoks et al., 2017). For the co-driver, there are no such HMIs visualizing the driver’s cognitive state. As a first step in this direction, a previous study (Perterer et al., 2015) investigated the benefits of a detailed navigation HMI for co-driver and driver. Besides a map overview, a satellite image, and turn-by-turn instructions, this HMI also displayed information about upcoming hazards. The participants stated that the main advantage of the HMI was that driver and co-driver had the same information about the route and could decide together how to react to certain situations, especially when they were dangerous. Therefore, more active involvement, as with an HMI, could be beneficial for both the co-driver and the driver, especially when they receive the same information. However, there is no scientific study directly investigating the influence of situational conditions on co-driver discomfort except for the survey by Innofact Ag for AutoScout24 (2013).

The co-driver’s estimation is also influenced by ***personal characteristics*** such as their experience as a co-driver or personality. These personal characteristics could influence how easily a situation is estimated as threatening. Ellinghaus and Schlag (2001) showed an influence of personal characteristics, like the experience as a co-driver and the attitude toward being a co-driver on the development of anxiety as a co-driver. The results indicated that more experienced co-drivers and co-drivers with a positive attitude toward their role experienced less anxiety. Participants who rarely are co-drivers and do not like it much were more afraid of accidents, false reactions of the driver, bad conditions of the car, and felt more bothered having no control as a co-driver. In general, previous studies also showed that people with higher ratings for neuroticism tend to experience stress or anxiety more likely (Gunthert et al., 1999; Jylhä and Isometsä, 2006). This could mean that co-drivers with higher loads on neuroticism could more likely experience situations as uncomfortable. Beggiato et al. (2015) showed that drivers with higher trust in automation requested less vehicle information about an automated vehicle during a ride. These results indicate that with higher trust it is less necessary to supervise automation and have means for control because they trust it to cope with future situations properly. For the co-driver this could mean that the more they trust the driver to handle situations properly, the less they feel exposed as a co-driver and need less additional information in order to “supervise” the driver. In contrast to these findings, some studies did not find an influence of personal characteristics on the development of discomfort. In one study, personal characteristics (e.g., Locus of control, Thrill and Adventure Seeking subscale) did not influence the experience of automated driving styles (Bellem et al., 2018). In the survey by Innofact Ag for AutoScout24 (2013), there was also no difference between women and men for the answers to the question of whether they have ever experienced an uncomfortable situation as a co-driver (79% women and 76% men stated “yes”). Although there exist studies that did not find an influence of personal characteristics on discomfort, most studies imply that the connection between them should be investigated.

Looking at the transactional model, the biggest difference between driver and co-driver is the limited possibility to ***cope*** with a situation. Since the co-driver has no access to vehicle controlling devices like pedals or the steering wheel there are only two different, indirect, ways for the co-driver to cope with the situation actively (problem-focused). One way would be requesting the driver to adapt their driving style to signal an uncomfortable situation. Another way would be to explicitly provide information about the criticality such as a too small distance. This could help the driver to realize the criticality of the situation leading to a feeling of own discomfort and an active coping to reduce it. For both types it means that social factors like the relationship or trust play a role. The co-driver must rely on the driver to also feel uncomfortable or at least to comprehend that such a situation can be uncomfortable for others. When the driver is reacting to the request, this could have a positive influence on the experienced discomfort and anxiety of the co-driver. When he/she is ignoring it, for example misunderstanding it as a criticism of his/her driving style, this could however also have no or an opposite effect. When active coping is not possible for the co-driver, the last coping strategy is the passive, emotion-focused way. The co-driver could try to calm down by distracting themselves, pushing an imaginary braking pedal, or by grabbing the door handle. It is possible that this passive way could also have a positive effect on their discomfort and anxiety (Strentz and Auerbach, 1988). If these coping strategies are not successful, the co-driver will feel exposed to the situation.

The previous paragraphs have shown the relevance of this topic and that there are hardly any scientific studies investigating conditions causing co-driver discomfort explicitly. The related work about driver discomfort can provide first insights into which conditions could be relevant for co-drivers. However, based on the transactional model in combination with a feedback-loop model, major additional differences between driver and co-driver regarding available information and coping might be relevant. Therefore, we have conducted an online questionnaire to provide a first overview of ***situational conditions*** of uncomfortable situations causing co-driver discomfort. We focused on the areas: ***“environment”, “vehicle”*,** and added the area ***“driver”*** possibly relevant for the co-driver. Additionally, we identified frequent co-drivers for additional more detailed interviews about uncomfortable situations as a co-driver through the questionnaire.

In order to propose an extended model describing the development of co-driver discomfort and ***coping***, the influence of ***personal characteristics, situational conditions, and situational characteristics***, as well as which role ***coping strategies*** play were considered in the detailed interviews. The results regarding these conditions and characteristics found in the questionnaire and interview are then used as the basis for discussing this extended model. The establishment of co-driver discomfort as a common problem and a proposed model of the cognitive origins enables future work to research means to reduce such negative feelings through technical interventions.

## Online Questionnaire

The survey of the opinion research institute Innofact Ag for AutoScout24 (2013) was so far the only work that directly considered co-driver discomfort. Besides the fact that we wanted to get a first overview of the topic, we also wanted to confirm these results with a scientific questionnaire and to go into more detail on certain points.

### Method

#### Participants

For the online questionnaire, participants of the Wuerzburg Institute for Traffic Sciences GmbH (WIVW) test panel (*N* = 730, living in the area of Wuerzburg, Germany, no selection criteria) were recruited via e-mail. In total, *N* = 119 (60% women, 40% men) people participated and completed the online questionnaire. The mean age of the sample was *m* = 41.28 years (*sd* = 15.77 years). Furthermore, approximately 59% of the sample reported being co-drivers 1-3 times a month or less, while the other 41% of the sample were weekly co-drivers (1-2 times a week until daily). Approximately 49% of the sample were daily drivers. The other 51% were driving 3-5 times a week or less.

#### Procedure

The survey was conducted using an online survey tool. This study was approved by the institutional ethics committee at the WIVW GmbH. This ethics committee follows recommendations of the German Research Association (Deutsche Forschungsgemeinschaft, 2019). Informed consent was obtained from each participant. Completing the online questionnaire took approximately 10 minutes per participant. The participants had to state if they had experienced any uncomfortable situations as a co-driver. In the introduction text, the difference between the concepts was detailed to avoid that participants confuse cognitive/psychological discomfort with discomfort as a symptom of motion sickness. We explained that the questionnaire is focused on cognitive/psychological discomfort as someone would experience in situations in which for example another vehicle would miss the presence of the own vehicle during a lane change or when the driver overtakes during poor visibility. Other forms of discomfort caused by motion sickness during a curvy ride or social discomfort caused by an impolite driver were explicitly discouraged to be considered for answers in the questionnaire. Afterward, the participants had to describe these situations via closed item format questions concerning driver type (e.g., family member, co-worker), frequency, and the reasons for discomfort (e.g., fast driving or weather) from the areas ***driver, vehicle, and environment***. The answers were presented in randomized order. The reasons for discomfort were requested for each stated driver type in order to allow for a dependent analysis. In the end, the participants could leave their e-mail address and consent that they could be contacted for a following detailed interview regarding co-driver discomfort.

### Results

The results showed that 88% of the participants have experienced at least one uncomfortable situation as a co-driver. The most frequently named driver type in uncomfortable situations was “family members/friends” (*n* = 97), followed with a larger gap by “coworkers/fellow students” (*n* = 40), “taxi drivers” (*n* = 18) and “driver of a lift” (*n* = 17). However, the rate of uncomfortable rides was the highest for “taxi drivers” (33% of participants named “more than 50% of rides”), followed by “co-worker/fellows” (25%), “driver of a lift” (18%) and smallest for “family members/friends” (14%). Table 1 shows the distribution of uncomfortable rides per driver group for male and female drivers. Since there is at least one cell with less than *N* = 5 in the driver groups “coworkers/fellow students,” “driver of a lift” and “taxi driver,” for these driver groups the Fischer‘s exact test was used. For the driver group “family members/friends” a chi-square test was executed. The tests revealed no significant differences between female and male participants regarding the amount of experienced uncomfortable rides for each driver group.

**TABLE 1:** Distribution of uncomfortable rides for female and male participants per driver group.

**Driver Group**	**Sex**	**Discomfort in less than 50% of rides**		**Discomfort in more than 50% of rides**		**Sum**		**χ*^2^***			
		***N***	**%**	***N***	**%**	***N***	**%**	**value**	**df**	***p***	**φ**
Family Members/Friends	Male	*49*	86	8	14	57	100	0.018	1	n.s.	−0.014
	Female	*34*	85	6	15	40	100				

								**Fischer’s Exact Test**			

										***p***	
Coworkers/Fellow Students	Male	*20*	69	9	31	29	100			n.s.	
	Female	*10*	91	1	9	11	100				
Driver of a Lift	Male	*8*	89	1	11	9	100			n.s.	
	Female	*6*	75	2	25	8	100				
Taxi Driver	Male	*9*	75	3	25	12	100			n.s.	
	Female	*3*	50	3	50	6	100				

*Note. n.s. = not significant for *p* > 0.05.*

**FIGURE 2:**
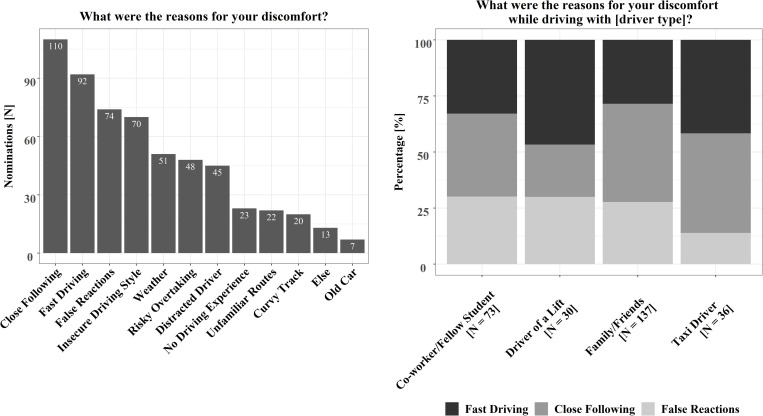
Reasons for co-driver discomfort (left). The three most mentioned reasons for co-driver discomfort by driver type (right).

Independent of the driver type, the most frequently experienced reasons were close following, fast driving, and false reactions (Figure 2, left). The pattern was similar when the reasons for discomfort were investigated by driver type. Except for situations with “driver of a lift,” the most frequently named reason for discomfort was close following, followed by fast driving and false reactions (Figure 2, right). Only for “driver of a lift” fast driving caused most of the co-driver’s discomfort.

### Discussion

The results of the online questionnaire show that uncomfortable situations as a co-driver are a common problem. There were no significant differences between female and male co-drivers in the experienced rates of uncomfortable rides for the different driver types. This also matches the findings of Innofact Ag for AutoScout24 (2013) which also showed no such differences. In general, the most frequently named reasons for discomfort were close following and fast driving. These reasons could be allocated to the factor “driver” and are characteristics of the driving style. The remaining reasons could be assigned to the factor “environment” (weather or unfamiliar routes) and the factor “vehicle” (old vehicle). When investigating the factors for discomfort depending on the four different driver types, the driving style (close following, fast driving, and false reactions) was again the most common cause of co-driver discomfort. The results indicate that the relationship to the driver might have an influence on how likely discomfort will be experienced but the reasons leading to discomfort, like driving style characteristics, were similar among the different driver types. A familiar driver might be assessed more easily. These results match the survey results of Innofact Ag for AutoScout24 (2013) in which the participants also named fast driving, close following, false reactions, and distraction of the driver as the main reasons for their discomfort. Furthermore, the results are also similar to the results of the study by Griesche et al. (2016) in which participants mentioned that they dislike small safety distances and high accelerations when driving with an automated vehicle. In the study by de Vos et al. (1997), participants also rated smaller distances as less comfortable during driving automated. However, the results differ from the survey results in the co-driver report by Ellinghaus and Schlag (2001). In this report, most participants named malfunctions of the vehicle as the main source of anxiety being a co-driver. A possible explanation could be that Ellinghaus and Schlag (2001) focused on anxiety and not discomfort. It is possible that false reactions of the driver are not perceived as negative as malfunctions of the vehicle. Such malfunctions of the vehicle may lead to more extreme emotions like anxiety because they are less frequent, and many people have little experience with the technical aspects of a vehicle. The frequently named reasons “close following” and “fast driving,” which are characteristics of a more offensive driving style, imply that defensive driving styles could reduce the co-driver’s discomfort. This is supported by the results of Yusof et al. (2016) or Mühl et al. (2019), which showed that defensive driving styles of automated vehicles are perceived as more comfortable.

The online questionnaire contained only closed questions investigating the influence of the three factors “***driver*,**” “***environment*,**” and “***vehicle***” on co-driver discomfort. The next step was to investigate uncomfortable situations through more detailed interviews. The online questionnaire results cannot provide information about the weight of the factors’ influence on co-driver discomfort and if there is a combination of reasons for discomfort or a single prominent factor. This fact was considered in the interviews through a rating regarding their influence on the co-driver. Furthermore, we considered the influence of ***personal characteristics*** on the development of co-driver discomfort.

## Interview

### Method

#### Participants

In this study, *N* = 24 participants from the online questionnaire sample (11 male and 13 female participants) with a mean age of *m* = 46.96 years (*sd* = 12.71 years) were interviewed. 65% of the participants were daily drivers, while 35% were drivers 3-5 times a week or less. 2/3 of the participants were weekly co-drivers, the other 1/3 of the sample were co-drivers 1 to 3 times a month.

#### Procedure

This study was approved by the institutional ethics committee at the WIVW GmbH. This ethics committee follows recommendations of the German Research Association (Deutsche Forschungsgemeinschaft, 2019). Informed consent was obtained from each participant. The interview had a duration of approximately 45 min per participant. It was semi-structured and consisted of a protocol with mixed open and closed questions. Similar to the online questionnaire, the different discomfort concepts were explained, and their differences were highlighted. Additionally, before the interview started, each participant was asked to explain to the experimenter their most recent uncomfortable situation and to explain why they experienced discomfort. This allowed the experimenter to check if the participant understood which type of discomfort was relevant for the interview. All situations described by the participants fulfilled these requirements. In the first part, participants answered questions about ***personal characteristics*** and they estimated how often they had experienced uncomfortable situations as a co-driver so far. They were asked about their attitude toward being a driver or a co-driver, if they feel exposed to the traffic, and if they prefer overview as a co-driver (6-point Likert scale 1 = “Does not apply at all” … 6 = “Totally agree”). In order to measure the personality trait neuroticism, the participants answered the item “Nervous” (“I easily get nervous and insecure”) on a 5-point Likert scale (1 = “Does not apply at all” … 5 = “Totally agree”). This item is part of the Big-Five-Inventory’s (BFI-10; Rammstedt and John, 2007) “Emotional Stability” scale.

In the second part, participants answered questions about ***situational conditions*** and characteristics of concrete uncomfortable situations. For this part, an adapted version of the critical incident technique (Flanagan, 1954) was selected. The participants were asked to recall their most recent uncomfortable situation as a co-driver and to give a short summary of this situation. Then the experimenter followed the structured interview. In this interview, the experimenter asked if ***situational conditions*** of the “***environment***”, “***vehicle***”, or “***driver***” influenced the uncomfortable situation. If conditions were relevant, the participants rated on a 6-point Likert scale (0 = “No influence at all” … 5 = “Very high influence”) how high the influence of these conditions on their discomfort was. They also answered questions about characteristics of the uncomfortable situation. They answered the following questions: how long the discomfort in the situation lasted, if they experienced discomfort in the situation on a 5-point Likert scale (1 = “Very little” … 5 = “Very strong”), if they experienced anxiety, if they felt exposed to the situation, if they trusted the driver, and if they assessed the situation as safety critical on a 6-point Likert scale (1 = “Does not apply at all” … 6 = “Totally agree”).

In the end, the participants named their ***coping strategies*** and if those strategies were helpful (“The chosen coping strategy was helpful to reduce my discomfort” rated on a 6-point Likert scale from 1 = “Does not apply at all” … 6 = “Totally agree”).

### Results

#### Personal Characteristics

The mean fraction of uncomfortable rides as a co-driver was about 20% (*median* = 10%). With a median = 3.00 the participants slightly disliked it to be a co-driver (Table 2). However, the results showed, that there was an almost even distribution of ratings across the entire scale, showing no clear tendency for this question. The dominant argument to dislike it was because they had no control as a co-driver, while the dominant argument for liking it was because it was relaxing. Most of the participants preferred to be the driver with a mean rating of *m* = 5.30 and the most frequently mentioned reason was that they liked it to drive on their own. As co-drivers, they preferred to keep an overview of the surrounding traffic in order to help or warn the driver (*m* = 4.50). They reported that they slightly feel exposed to traffic as a co-driver with a mean rating of *m* = 3.83 because they could not intervene in the situation. On the other side, some participants fully trusted the driver.

**TABLE 2:** Distribution of ratings for personal characteristics (Item) and named reasons for ratings by participants.

		**Disagree ≤ 3**			**Agree ≥ 4**		
**Item**		**Reason**	***n***	***N***	**Reason**	***n***	***N***
“I Like it to be a co-driver”	*m* = 3.42 *sd* = 1.74	”I have no control”	8	14	”It’s relaxing”	5	10
					”I trust the driver”	2	
		”I do not like the driving style”	4		”I still like driving”	2	
		Other	2		Other	1	
”I like it to be a driver”	*m* = 5.30 *sd* = 0.97	”I’m not a car fan”	2	2	”It’s fun/I like driving”	15	21
					”I have control”	6	
”I prefer overview as a co-driver”	*m* = 4.50 *sd* = 1.53	”I trust the driver”	3	5	”I want overview to help/warn driver”	10	19
		Other	2		”I want control”	2	
					Other	7	
”I feel exposed as a co-driver”	*m* = 3.83 *sd* = 1.58	”I trust the driver”	4	9	”I cannot intervene”	12	15
		”Depends on driving style of driver”	3		”I trust the driver”	1	
		Other	2		Other	2	

*Note. m, mean; sd, standard deviation.*

The results showed no significant relations of co-driver’s personal characteristics to the rated discomfort in the situation (Table 3).

**TABLE 3:** Correlations of personal characteristics of the co-driver with their experienced discomfort in the situation.

**Variables (*N* = 24)**	**Discomfort**	**Sig. (2-tailed)**
Sex	Point-Biserial: *r* = −0.23	n.s.
Age	Spearman’s rho*: r* = −0.01	n.s.
Experience as Co-Driver	Spearman’s rho: *r* = −0.01	n.s.
Nervous (BFI)	Pearson: *r* = 0.14	n.s.
“I Like it to be a co-driver”	Pearson: *r* = −0.17	n.s.
“I prefer overview as a co-driver”	Pearson: *r* = −0.31	n.s.
“I feel exposed as a co-driver”	Pearson: *r* = 0.02	n.s.

*Note. n.s. = not significant for *p* > 0.05.*

#### Situational Conditions and Characteristics of Uncomfortable Situations

**FIGURE 3:**
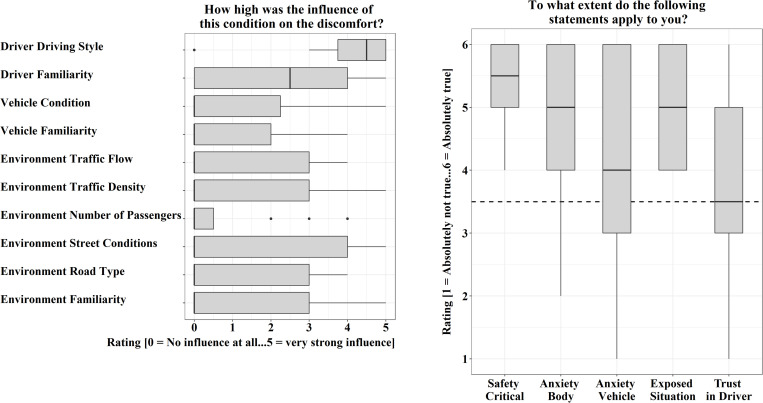
Situational conditions of the three areas and ratings regarding their influence on the discomfort (left). The four participants which named reasons of the area environment and vehicle and all situational conditions which were rated by more than 75% of the participants as “not influential” (rating = 0) are not presented in the graph. Rated characteristics of their last uncomfortable situations (right).

The most recent situations recalled by the participants were perceived as very uncomfortable as reflected in a mean discomfort rating of *m* = 4.00 (*sd* = 1.38). In these uncomfortable situations, the driver was most often a family member or a friend (approximately 79%). The remaining 21% of cases were with drivers of a lift or of a taxi. The participants also specified how long the feeling of discomfort lasted and 67% explained that the uncomfortable feeling was limited to a part of the route or a special situation, followed by 21% feeling uncomfortable during the complete route and 12% during the complete route and afterward. Furthermore, in 20 of the 24 named uncomfortable situations, the “***driver***” was the main factor causing the co-driver’s discomfort. In Figure 3 on the left, the ratings of these 20 participants are displayed. They rated the driving style as highest in influence on their discomfort with a mean rating of *m* = 4.10 (*sd* = 1.25), in particular high velocities, close following, and an aggressive driving style. Of the remaining uncomfortable situations, three were caused by conditions of the “***environment.***” In these three situations, conditions of the “***environment road type*,**” especially differing conditions of traffic and infrastructure in the city, on the autobahn, or rural roads were rated as high in their influence. One situation was caused by conditions of the factor “***vehicle***” (malfunction of the gas pedal). Each one of these *N* = 4 participants rated the driving style as not influential (rating = 0). Uncomfortable situations were perceived as clearly safety critical (*m* = 5.38, *sd* = 0.71) and the participants were afraid of negative consequences like damages (*m* = 4.21, *sd* = 1.67) and injuries (*m* = 4.83, *sd* = 1.20) (Figure 3 right). Furthermore, the participants felt exposed to the situation (*m* = 5.08, *sd* = 0.83). Their trust in the driver was neither clearly high nor very low (*m* = 3.79, *sd* = 1.53).

Correlation analysis (Table 4) indicates that with increasing discomfort situations were rated as more safety critical (*r* = 0.49), participants experienced more anxiety toward injuries (*r* = 0.43) or damage to the vehicle (*r* = 0.42), and they felt more exposed to the situation as a co-driver (*r* = 0.56). They also felt more exposed to the situation, when their trust in the driver is reduced (*r* = −0.43).

**TABLE 4:** Pearson correlations of characteristics of uncomfortable situations.

**Characteristics (*N* = 24)**	**Discomfort**	**Safety Critical**	**Anxiety Body**	**Anxiety Vehicle**	**Exposed Situation**
Safety Critical	*r* = 0.49*	–	–	–	–
Anxiety Body	*r* = 0.43*	*r* = 0.43*	–	–	–
Anxiety Vehicle	*r* = 0.42*	*r* = 0.52*	*r* = 0.60*	–	–
Exposed Situation	*r* = 0.56*	*r* = 0.39	*r* = 0.36	*r* = 0.68*	–
Trust in Driver	*r* = 0.02	*r* = −0.12	*r* = 0.00	*r* = −0.12	*r* = −0.43*

Note. Significant (*p* < 0.05) correlations are marked with *.

#### Coping Strategies

**FIGURE 4:**
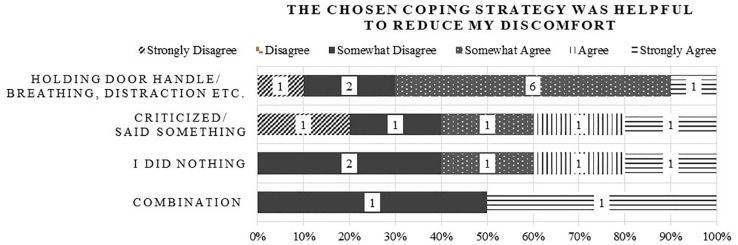
Frequencies of named coping strategy to reduce co-driver discomfort and ratings how helpful they were.

The participants were asked to name coping mechanisms they used to reduce their uncomfortable feelings (Figure 4). Most of the participants (*N* = 10) named emotion-focused coping behavior like holding the door handle, deep breathing, or distraction, followed by *N* = 5 who said something to the driver or criticized him/her (problem-focused coping), *N* = 2 who combined the above strategies, and *N* = 5 who did nothing. The ratings of the coping mechanism were very mixed showing only a slight tendency of being helpful to reduce their discomfort. *N* = 2 participants could not show any coping strategies as the situation was too sudden.

### Discussion

The results show that uncomfortable feelings occur most often during specific situations rather than for a whole ride. The driving style of the driver influences whether a situation is perceived as safety critical and therefore causes discomfort and anxiety in the co-driver. More safety critical situations lead to higher amounts of experienced discomfort and anxiety. If co-drivers feel more exposed to the situation, this can also increase the experienced discomfort and anxiety. This feeling of being exposed can increase if the trust in the driver is low. In the interview, the explanations provided by the participants concerning the statement “I feel exposed as a co-driver” support this conclusion. If they agreed, many participants mentioned that they feel exposed because they cannot intervene, the ones that disagreed explained it with their low trust in the driver. This indicates that both the presence of a situation perceived as safety critical and the missing possibility to actively cope with it can lead to a feeling of being exposed to the situation and consequently have an influence on co-driver discomfort and on anxiety of negative outcomes. It is possible that the definition of discomfort explained to the participants in the introduction primed more driving style related answers. However, it is important to note that besides this fact, participants mentioned that it also has an important influence on their discomfort that they feel exposed as a co-driver.

The participants also mentioned how they handled uncomfortable situations and named either strategies focused on changing emotions (e.g., “grasping the door handle”, “distraction”) or strategies focused on changing the situation (e.g., “asking the driver to keep more distance”). However, the different types of coping strategies were perceived neither clearly helpful nor not helpful in reducing their discomfort. Even the most direct strategy of “asking the driver to keep more distance,” seemed only partially effective. This could be caused by drivers who do not follow the requests which could in turn have a negative influence on the trust in the driver.

In contrast to Ellinghaus and Schlag (2001), the influence of ***personal characteristics*** like experience as co-driver or the attitude toward being a co-driver on the evaluation of a situation could not be found. One explanation could be that they focused on co-driver anxiety which is a more intense emotion than discomfort. Therefore, it is possible that this focus of the questions produced a greater difference in the groups and answers. They also took their conclusions based on differences in the frequencies of the groups and not based on statistical tests. In contrast to research on motion sickness, we did not find correlations of discomfort with sex (Koslucher et al., 2015). The results of the present interview however correspond to the findings by Innofact Ag for AutoScout24 (2013) which also showed no difference between sexes. This indicates that cognitive/psychological discomfort investigated in this work is different from experienced discomfort during motion sickness. Bellem et al. (2018) similarly found no correlation between relatively general personal characteristics like Locus of Control or Sensation Seeking and the preferred driving style of an automated vehicle. They just found a small relationship between the participants’ own driving style and the preferred automated driving style. However, as participants selected very different uncomfortable situations in the interviews, it would be interesting to investigate the effect of personal characteristics if they would all experience similar situations.

## Model of Causes for Co-Driver Discomfort

**FIGURE 5:**
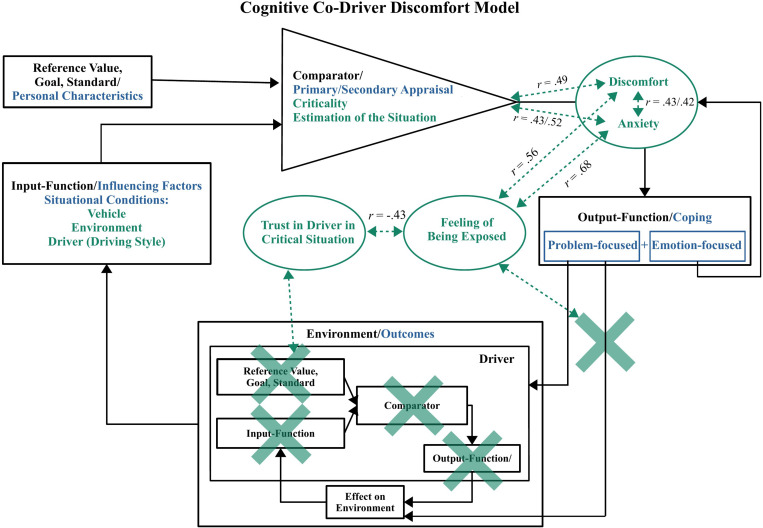
Co-Driver Discomfort Model describing the development of co-driver discomfort considering limited information about the cognitive state of the driver and limited control over the situation. Elements of the transactional model that are adapted from Lazarus and Folkman (1984) are marked in blue. The elements and causal connections of the feedback-loop model adapted from Carver and Scheier (1998) are shown in black. Additional components and respective correlations found in the interview and questionnaire are drawn in green with dashed arrows. Aspects that are not available or have limited accessibility to a co-driver are crossed out.

Based on the literature and the presented results it is possible to develop an extended model, describing why co-drivers experience discomfort. The driver’s feedback-loop in Figure 5 below shows the driver’s regulation and subjective estimation process (comparator) based on ***personal characteristics*** and ***situational conditions***, like actual velocity and distance (input-function), with his/her preferred velocity and distance (reference value). However, as Figure 5 is modeling the cognitive processes of the co-driver, all parts of the driver’s cognitive state in his/her feedback loop that are not accessible to the co-driver are crossed out. As mentioned in the introduction, it can be assumed that the comparison process within the feedback loop of the co-driver is also based on the same ***personal characteristics*** and ***situational conditions*** (Figure 5 top left). The major difference between the driver’s and co-driver’s feedback-loop is the fact that the co-driver does not have the possibility to change the actual velocity or distance with a direct action such as braking. There are discrepancies between the amount of information available to driver and co-driver such as limited information about the cognitive state of the driver or a different perspective. This can cause a different estimation of a situation’s criticality. This can also lead to the consequence that some situations, which are objectively not safety critical, are evaluated as safety critical by the co-driver. This is supported by the assumption that very few drivers would voluntarily keep an uncomfortable driving style or a driving style that they consider themselves as safety critical, while it might still cause co-driver discomfort. The study by Yusof et al. (2016) showed that assertive drivers did not prefer their own driving style in automated vehicles. Different roles in a vehicle and subsequently different amounts of available information and control can lead to a different estimation of driving styles or situations.

Such safety critical situations can then cause a feeling of discomfort which can increase as the situation becomes more critical. This is supported by the strong relationship found in the interviews (*r* = 0.49). As the definition for discomfort by the Oxford English Dictionary (Stevenson and Lindberg, 2017) indicates, discomfort can be accompanied by anxiety in such an alleged critical situation (*r* = 0.43 for anxiety regarding injuries and *r* = *0.42* regarding vehicle damages). After the evaluation of the situation, co-drivers try to ***cope*** with the situation in either an active or passive way (Lazarus and Folkman, 1984). The majority of the participants used one or both of these strategies to cope with their discomfort. Most of them showed more passive emotion-focused strategies. One explanation could be that these strategies are preferred because they are less offensive and avoid conflicts with the driver. All coping strategies showed very mixed helpfulness ratings. Even the problem-focused coping strategy which could directly influence the discomfort causing factor ***driver*** showed mixed helpfulness ratings. One reason could be that when the driver is the causing factor and the co-driver asks for an adaption of the driving style, he/she must trust the driver that he/she will follow the request. This can limit the co-drivers feeling of having control in the situation. The mixed helpfulness ratings of this coping strategy indicate that there can be drivers ignoring this request.

This situation of being limited to actively ***cope*** with the situation or an unsuccessful attempt to cope with it can lead to a feeling of being exposed as a co-driver. This conclusion is not only supported by the relationship between their rated trust in the driver and the co-drivers feeling of being exposed in the situation (*r* = −0.43) found in the interview, but also by the answer that they only feel exposed in the mentioned uncomfortable situations and do not generally feel exposed as a co-driver. The results by Beggiato et al. (2015) support the considerations made in the model that trust in the system/driver can have an influence on how much control and information you want to receive. With higher trust in automation, the participants requested less information to supervise the system and engaged more in secondary tasks, if there was no complex situation. These results indicate that co-drivers could have a higher need for information about the driver’s cognitive state and a higher need for more means of active control when the trust in the driver is reduced since they feel more exposed in these uncomfortable situations. The feeling of being exposed can then in turn increase their already existing discomfort and fear. This is also supported by the relationships found in the interviews for these characteristics (*r* = 0.56 for feeling exposed and discomfort; *r* = *0.68* feeling exposed and anxiety).

## General Discussion

The results of the online questionnaire indicate that co-driver discomfort seems to be a frequently occurring problem. More frequent co-drivers feel uncomfortable in about every 5th ride. The work in this paper provides evidence that co-drivers should also be considered in the design and evaluation of passenger vehicles. The questionnaire established co-driver discomfort as a common issue and together with the detailed interviews, shed light on possible causes. The driving style of the driver, especially close following and driving at high velocities, could be identified as an influencing factor with regard to whether co-drivers perceived situations as safety critical and therefore experienced discomfort. This is in line with the results of the Innofact Ag for AutoScout24 (2013) and other studies (de Vos et al., 1997; Griesche et al., 2016; Mühl et al., 2019). In the online questionnaire, the interview and previously in the public survey by Innofact Ag for AutoScout24 (2013) characteristics of the vehicle, like its condition, were rarely mentioned, contrary to the results of Ellinghaus and Schlag (2001). Although malfunctions of the car might also cause discomfort, they occur less frequently, which might limit their overall impact on the reports. Despite the smaller sample in the online questionnaire the results of the representative survey of the Innofact Ag for AutoScout24 (2013) could be confirmed. As the online questionnaire was more detailed than this survey, additional results could be found. Besides the driving style, the participants mentioned another main factor leading to co-driver discomfort. This factor was their limited possibility to actively cope with the situation or respectively their lack of control leading to a feeling of being exposed. This is indicated by more frequently used emotion-focused coping strategies and the overall mixed helpfulness ratings of the emotion-focused and even the problem-focused coping strategies.

The results of the two studies were used to propose a cognitive model that can explain some of the relations and provide opportunities for future detailed investigations. The model is based on two well-established theories (feedback-loop model and transactional stress model) and extends them to a dyadic co-driver-driver system. The model includes the finding that besides the driving style of the driver, a feeling of missing control or being exposed, caused by the limited possibilities of the co-driver to cope with the situation, can influence co-driver discomfort. In future research, the developed model can be tested by investigating the influence of additional information about the driver’s attention or opportunities of active control for the co-driver.

## Practical Implications

The results of this work and the developed model could act as a basis for the design of a “co-driver assistant system” showing information or providing control in order to reduce the co-driver’s discomfort. Each of the identified aspects of missing information or interaction provides an opportunity for technological intervention. Such interventions can mean that an uncomfortable situation can be turned into a neutral or comfortable one by reducing the perceived criticality. Furthermore, with certain additional information, the co-driver could have a positive effect since he/she could support the driver with the driving task. Such a positive effect of co-driver support was also found in the study by Perterer et al. (2015) and the positive effect of co-driver presence on driving safety was supported by Vollrath et al. (2002). Besides an increase of co-driver comfort such an assistant system could also make it more attractive being a co-driver. The results are also relevant for the development of interfaces in highly automated vehicles when drivers will partially turn to passengers. However, assistance in manual vehicles has additional difficulties when it comes to gathering information about a human driver’s internal states. It will therefore be interesting to further research the level of granularity that is sufficient to lower co-driver discomfort in a real vehicle.

## Data Availability Statement

The raw data supporting the conclusions of this article will be made available by the authors, without undue reservation.

## Ethics Statement

The studies involving human participants were reviewed and approved by our own ethics committee following a strict ethics protocol when planning our studies with healthy participants. This ethics committee follows recommendations of the German Research Association (Deutsche Forschungsgemeinschaft, 2019). The patients/participants provided their written informed consent to participate in this study.

## Author Contributions

SI, DM, and TW did the conceptualization, methodology, validation, and resources. SI did the software, formal analysis, investigation, data curation, writing – original draft preparation, and visualization. DM and TW did the writing – review and editing. SI and TW did the project administration. Supervision and funding acquisition, not relevant. All authors have read and agreed to the published version of the manuscript.

## Conflict of Interest

This study was conducted as part of a research program of the Honda Research Institute Europe GmbH. TW is employee of this company. He contributed to the design of the study, review and editing of the manuscript and the decision to publish the results. The authors SI and DM were also employed by Wuerzburg Institute for Traffic Sciences GmbH.
