# Corrigendum: The Discomfort of Riding Shotgun – Why Many People Don't Like to Be Co-driver

**DOI:** 10.3389/fpsyg.2021.696426

**Published:** 2021-05-27

**Authors:** Sandra Ittner, Dominik Mühlbacher, Thomas H. Weisswange

**Affiliations:** ^1^Wuerzburg Institute for Traffic Sciences GmbH, Veitshöchheim, Germany; ^2^Honda Research Institute Europe GmbH, Offenbach, Germany

**Keywords:** information processing, cognition, passenger, comfort, feedback-loop model, situation awareness, risk assessment, autonomous driving

In the original article, there was a mistake in Table 4 as published. In the published article, all entries were marked as having significant correlations, although this was only true for a subset. However, the values in the table and their discussion in the article were stated correctly and therefore this correction does not change the content or conclusions of the article. The corrected Table 4 appears below.

**Table 4:** Pearson correlations of characteristics of uncomfortable situations.

**Characteristics (*N* = 24)**	**Discomfort**	**Safety Critical**	**Anxiety Body**	**Anxiety Vehicle**	**Exposed Situation**
Safety Critical	*r* = 0.49*	–	–	–	–
Anxiety Body	*r* = 0.43*	*r* = 0.43*	–	–	–
Anxiety Vehicle	*r* = 0.42*	*r* = 0.52*	*r* = 0.60*	–	–
Exposed Situation	*r* = 0.56*	*r* = 0.39	*r* = 0.36	*r* = 0.68*	–
Trust in Driver	*r* = 0.02	*r* = −0.12	*r* = 0.00	*r* = −0.12	*r* = −0.43*

Note. Significant (p < 0.05) correlations are marked with ^*^.

The authors apologize for this error and state that this does not change the scientific conclusions of the article in any way. The original article has been updated.

